# A new *Toxoplasma gondii* chimeric antigen containing fragments of SAG2, GRA1, and ROP1 proteins—impact of immunodominant sequences size on its diagnostic usefulness

**DOI:** 10.1007/s00436-015-4552-6

**Published:** 2015-06-09

**Authors:** Bartłomiej Ferra, Lucyna Holec-Gąsior, Józef Kur

**Affiliations:** Department of Molecular Biotechnology and Microbiology, Faculty of Chemistry, Gdańsk University of Technology, Narutowicza 11/12, 80-233 Gdańsk, Poland

**Keywords:** ELISA, Serological detection, Chimeric antigen, SAG2, GRA1, ROP1

## Abstract

This study presents the first evaluation of new *Toxoplasma gondii* recombinant chimeric antigens containing three immunodominant regions of SAG2, GRA1, and one of two ROP1 fragments differing in length for the serodiagnosis of human toxoplasmosis. The recombinant chimeric antigens SAG2-GRA1-ROP1_L_ (with large fragment of ROP1, 85–396 amino acid residues) and SAG2-GRA1-ROP1_S_ (with a small fragment of ROP1, 85–250 amino acid residues) were obtained as fusion proteins containing His_6_-tags at both ends using an *Escherichia coli* expression system. The diagnostic utility of these chimeric antigens was determined using the enzyme-linked immunosorbent assay (ELISA) for the detection of specific anti-*T. gondii* immunoglobulin G (IgG). The IgG ELISA results obtained for the chimeric antigens were compared to those obtained for the use of *Toxoplasma* lysate antigen (TLA) and for a mixture of recombinant antigens containing rSAG2, rGRA1, and rROP1. The sensitivity of the IgG ELISA was similar for the SAG2-GRA1-ROP1_L_ chimeric antigen (100 %), the mixture of three proteins (99.4 %) and the TLA (97.1 %), whereas the sensitivity of IgG ELISA with the SAG2-GRA1-ROP1_S_ chimeric antigen was definitely lower, reaching 88.4 %. In conclusion, this study shows that SAG2-GRA1-ROP1_L_ chimeric antigen can be useful for serodiagnosis of human toxoplasmosis with the use of the IgG ELISA assay. Therefore, the importance of proper selection of protein fragments for the construction of chimeric antigen with the highest reactivity in ELISA test is demonstrated.

## Introduction

Toxoplasmosis is a widespread disease caused by the obligate intracellular parasite *Toxoplasma gondii*, which can infect a wide range of hosts, including humans (Tenter et al. 2000). The World Health Organization (WHO) calculated that one third of the human population is infected with *T. gondii*. In healthy individuals, the primary infection is generally asymptomatic or causes relatively mild flu-like symptoms. However, the disease is a major threat to immunocompromised individuals and pregnant women. For immunocompromised patients, infection with *T. gondii* may cause serious problems, such as fever, headache, encephalitis, pneumonia, myocarditis, conjunctivitis, and nervous system damage (Ambroise-Thomas 2001). In pregnant women, toxoplasmosis may pose serious problems because of transplacental transmission which can cause fetal abortion. The primary infection can also lead to premature birth of fetus, neonatal malformation, neurological damage, and blindness (Dunn et al. 1999; Fatoohi et al. 2002).

Currently, the assays for the diagnosis of toxoplasmosis primarily use an extract of whole *T. gondii* tachyzoites grown in mice or tissue cultures. Although the TLA is characterized by high sensitivity and specificity in the immunoassay tests, long and expensive procedures that require a parasite culture are the biggest disadvantage of the production of the *T. gondii* lysate. An additional drawback may be a problem with the standardization of the tests, which is a direct result of the quality of the obtained TLA. This is correlated with differences in culturing procedures and methods of lysate preparation between individual laboratories. Moreover, in some cases, the results obtained for commercial tests are ambiguous, which does not allow correct diagnosis. Unfortunately, the appropriate diagnosis can be crucial, especially for pregnant women or any immunocompromised person. For these reasons, intensive research of recombinant antigens and their mixtures to replace the TLA in ELISA tests is being carried out. The usefulness of the recombinant antigens and/or mixture of several recombinant proteins in the detection of specific IgG and IgM anti-*T. gondii* antibodies is broadly evidenced (Holec-Gąsior 2013). In recent years, a completely new approach has been proposed with the use of recombinant chimeric antigens containing different immunoreactive regions from several selected *T. gondii* antigens. Until now, there were only a few studies demonstrating the usefulness of the recombinant chimeric antigens in the detection of specific anti-*T. gondii* antibodies in human sera (Beghetto et al. 2006; Dai et al. 2012, 2013; Holec-Gąsior et al. 2012a, b; Lau et al. 2011).

The aim of the present study is the evaluation of a new SAG2-GRA1-ROP1 chimeric antigen for ELISA tests for serodiagnosis of human toxoplasmosis and demonstration of the importance of proper selection of protein fragments for the construction of the chimeric antigen with the highest reactivity in the ELISA test. Selection of these antigens for the construction of chimeric protein was based on earlier results obtained in the immunoassays, which determined the reactivity of these proteins with specific anti-*T. gondii* antibodies. The SAG2 antigen, which is identified as an intrinsically unstructured protein, can interact with many cellular and surface molecules of infected host (Macedo et al. 2013). In previous studies, the usefulness of the recombinant SAG2 antigen was shown to be effective in detecting specifically anti-*T. gondii* antibodies in sera especially from the acute, but also from the chronic phase of toxoplasmosis (Hiszczyńska-Sawicka et al. 2005; Lau and Fong 2008; Li et al. 2000; Parmley et al. 1992). The GRA1 antigen, which plays an important role in the structural modifications of parasitophorous vacuole, is also associated with strong stimulation of the host immune system (Cesbron-Deleuw et al. 1989; Coppens et al. 1999). In GRA1, the immunodominant epitope involved in the human B-cell response against the parasite was identified and this suggests that GRA1 antigen can be used as a marker of the chronic phase of toxoplasmosis (Cesbron-Deleuw et al. 1989; Beghetto et al. 2001). Serological studies with recombinant GRA1 antigen demonstrated that this protein can be used to detect specific IgG in sera of both acute and chronic phase of disease (Hiszczyńska-Sawicka et al. 2003; Lecordier et al. 2000; Pietkiewicz et al. 2007). The ROP1 protein is involved at an early stage of the parasite invasion into host cells. The evidence of this is the fact that ROP1 is secreted into the interior of the forming parasitophorous vacuole during parasite entry into host cells, and its expression is inhibited a few hours after the invasion (Bradley et al. 2002; Soldati et al. 1998). For this reason, the ROP1 protein is considered to be a marker for the differentiation between acute and chronic toxoplasmosis, which has been confirmed in immunoassays (Holec-Gąsior et al. 2009, 2010).

## Materials and methods

### Construction of the recombinant plasmid

The pUET1/SAG2 (Hiszczyńska-Sawicka et al. 2005), pUET1/GRA1 (Hiszczyńska-Sawicka et al. 2003), and pUET1/ROP1 (Holec-Gąsior et al. 2009) recombinant plasmids were used as templates for amplification of correct DNA fragments using a standard PCR amplification protocol with the Delta3 DNA polymerase (BLIRT S.A., Gdansk, Poland). DNA fragments of *sag2*, *gra1*, *rop1*_*S*_, and *rop1*_L_ were amplified using S1, S2, G1, G2, R1, R2, and R3 primers (Table 1). PCR products of *sag2* and *gra1* were then mixed and used as templates in a PCR with S1 and G2 primers. *sag2*/*gra1* and *rop1*_S_ and *rop1*_L_ products were then mixed and used as templates in a PCR with the primers pairs S1 + R2 and S1 + R3, which were designed to contain *Bgl*II and *Eco*RV recognition sequences to facilitate cloning. The final PCR products were digested with both *Bgl*II and *Eco*RV and inserted into the *Bgl*II and *Eco*RV sites of the pET-30 Ek/LIC vector (Novagen, Madison, WI, USA). The nucleotide sequences of the resulting recombinant plasmids were confirmed by DNA sequencing (Genomed, Poland). The pET-30/SAG2-GRA1-ROP1_S_ and pET-30/SAG2-GRA1-ROP1_L_ plasmids contained a sequence of SAG2, amino acid residues 31–170, a sequence of GRA1, amino acid residues 26–190, and a sequence of ROP1, amino acid residues 85–250 and 85–396, which were embedded in a frame between the His_6_-tag domains for purification of the recombinant proteins by means of metal affinity chromatography.

**Table 1:** Oigonucleotide primers used for construction of the SAG2-GRA1-ROP1_S_ ant the SAG2-GRA1-ROP1_L_ chimeric antigens

*T. gondii* gene(s)	Primer name	Primer sequence	Underlined sequence	Template for amplification
*sag2*	S1 (forward)	5′-GACAGCACAGATCTGACGCCAGCGCCCATTG-3′	*Bgl*II and fragment of *sag2*	pUET1/SAG2
	S2 (reverse)	5′-GTTGTCGCCGCCTTCCGTGAGAGACACAGG-3′	Fragments of *gra1* and *sag2*	
*gra1*	G1 (forward)	5′-CCTGTGTCTCTCACGGAAGGCGGCGACAAC-3′	Fragments of *sag2* and *gra1*	pUET1/GRA1
	G2 (reverse)	5′-CGGGCCTCTGACAGGCTCTCTCTCTCCTG-3′	Fragments of *rop1* and *gra1*	
*rop1*_*S*_	R1 (forward)	5′-CAGGAGAGAGAGAGCCTGTCAGAGGCCCG-3′	Fragments of *gra1* and *rop1*	pUET1/ROP1
	R2 (reverse)	5′-CGGATCCGATATCGCACGACCTGGTCCCTGC-3′	*Eco*RV and fragment of *rop1*	
*rop1*_*L*_	R1 (forward)	5′-CAGGAGAGAGAGAGCCTGTCAGAGGCCCG-3′	Fragments of *gra1* and *rop1*	pUET1/ROP1
	R3 (reverse)	5′-CGGCTCCGATATCGCTTGCGATCCATCATCCTG-3′	*Eco*RV and fragment of *rop1*	
*sag2*/*gra1*	S1 (forward)	Same as above	Same as above	*pre*-*sag2*/*gra1*
	G2 (reverse)	Same as above	Same as above	
*sag2*/*gra1*/*rop1*_*S*_	S1 (forward)	Same as above	Same as above	*pre*-*sag2*/*gra1*/*rop1*_*S*_
	R2 (reverse)	Same as above	Same as above	
*sag2*/*gra1*/*rop1*_*L*_	S1 (forward)	Same as above	Same as above	*pre*-*sag2*/*gra1*/*rop1*_*L*_
	R3 (reverse)	Same as above	Same as above	

### Expression and purification of the chimeric antigens and recombinant proteins

*E. coli* strain Rosetta(DE3)pLacI, transformed with pET-30/SAG2-GRA1-ROP1_S_ or pET-30/SAG2-GRA1-ROP1_L_ recombinant plasmid, was grown 16 h at 30 °C in LB media supplemented with 100 μg/ml of ampicillin and 34 μg/ml of chloramphenicol. Following day, 1000 ml of LB medium, supplemented with the same antibiotics, was inoculated with 20 ml of the overnight culture. The cultures were grown with vigorous shaking at 30 °C to the optical density at 600 nm (OD_600_) of 0.4. Protein production was then induced with isopropyl-β-d-thiogalactopyranoside (IPTG) to a final concentration of 1 mM, and the cells were incubated with vigorous shaking for an additional 18 h at 30 °C. Cells were then harvested by centrifugation, and the pellets were resuspended in 30 ml of buffer A (20 mM Tris–HCl pH 7.9, 500 mM NaCl, 5 mM imidazole, 0.1 % Triton X-100). The cells were disrupted by sonication, and the insoluble debris was removed by centrifugation. The protein was purified from the supernatant using a Ni^2+^-iminodiacetic acid-Sepharose column in accordance with the manufacturer’s instructions (Novagen, Madison, WI, USA).

Recombinant proteins SAG2 (from 31 to 170 amino acid residues), GRA1 (from 26 to 190 amino acids), and ROP1 (from 85 to 396 amino acid residues) were produced as previously described (Hiszczyńska-Sawicka et al. 2003, 2005; Holec-Gąsior et al. 2009). TLA was prepared from tachyzoites (RH strain) as described earlier (Holec-Gąsior et al. 2009). The recombinant proteins were analyzed by means of SDS-PAGE on 12 % acrylamide gels and stained with Coomassie blue. The concentrations of the purified proteins were determined by the Bradford method using bovine serum albumin as the standard.

### Human serum samples

All of the serum samples used in this study were received during routine toxoplasmosis screening. The samples were previously collected with prior informed consents of the individuals. Approval for use of the banked samples was obtained from Medical University of Gdańsk Human Research Ethics Committee. A total of 295 sera were analyzed and divided into four groups in accordance with the results obtained using the commercial tests VIDAS TOXO IgM, VIDAS TOXO IgG II, and VIDAS TOXO IgG AVIDITY (bioMérieux, Marcy l'Etoile, France). Group I serum samples were collected from 41 patients with suspected acute phase of toxoplasmosis. All of these sera had specific IgM and IgG antibodies with low avidity. Group II consisted of 17 serum samples received from patients with suspected postacute phase of toxoplasmosis. Selection was based on the presence of specific IgG antibodies with low or borderline avidity and an absence of specific IgM antibodies. Group III included 114 sera from patients with chronic toxoplasmosis. All of these serum samples had IgG antibodies with high avidity and specific IgM antibodies were not detected. Moreover, the serum samples from this group were further divided into three subgroups based on IgG titers: IIIA consisted of 17 serum samples with high value of IgG antibodies (over 300 IU/ml), IIIB included 34 serum samples with values of IgG antibodies between 101 to 300 IU/ml, and IIIC consisted of 63 serum samples with low values of IgG antibodies below 100 IU/ml. The last group, IV, was a control group of 123 human serum samples from seronegative patients.

### IgG ELISA

MaxiSorp multiwell plates (Nunc, Roskilde, Denmark) were coated with chimeric antigens SAG2-GRA1-ROP1_S_, SAG2-GRA1-ROP1_L_, and a mixture of proteins (rSAG2, rGRA1, and rROP1) or with TLA at final concentrations of 2.5 μg/ml for each recombinant protein and 1 μg/ml for the TLA in a coating buffer (0.05 M carbonate buffer, pH 9.6). After overnight incubation at 4 °C, the plates were washed three times with PBS-0.1 % Triton X-100 and blocked for 1 h at 37 °C in blocking solution (1 % bovine serum albumin, 0.5 % Triton X-100 in PBS). The cells were then washed three times and incubated for 1 h at 37 °C with the human serum diluted 1:100 in blocking solution. Next, the plates were washed three times with washing buffer and incubated with anti-human IgG peroxidase-labeled conjugates (Jackson ImmunoResearch, Newmarket, Suffolk, UK) diluted 1:16,000 in blocking solution for 1 h at 37 °C, after which *o*-phenylenediamine dihydrochloride chromogenic substrate (Sigma-Aldrich, Dorset, UK) was added. After 45 min of incubation at 37 °C in darkness, the reaction was stopped by the addition of 0.1 M sulfuric acid and the OD_492_ was measured using a microtiter plate reader (Multiskan FC; Thermo Scientific).

Each serum sample was examined twice. The results were determined for each sample by calculating the mean OD reading of duplicate wells. A positive result was defined as any value higher than the average OD reading plus 2 standard deviations (cutoff) obtained with 23 serum samples from the control, group IV, which consisted of seronegative serum samples. The calculated cutoff values were 0.281 for the SAG2-GRA1-ROP1_S_, 0.389 for rSAG2-GRA1-ROP1_L_, 0.504 for SAG2, GRA1, and ROP1 mixture, and 0.507 for TLA.

## Statistical analysis

Means and range of the IgG ELISA absorbance measurements for the sera from different groups, and the sensitivity, specificity, and positive and negative predictive values of IgG ELISA assays are given.

## Results

### Expression and purification of the SAG2-GRA1-ROP1_S_ and SAG2-GRA1-ROP1_L_ chimeric antigens

The SAG2-GRA1-ROP1_S_ and SAG2-GRA1-ROP1_L_ were expressed as soluble proteins with a calculated molecular mass of 56.4 and 72.2 kDa, respectively. The proteins were purified by a one-step chromatography procedure by metal affinity chromatography with Ni^2+^ bound to iminodiacetic acid-agarose (Novagen). The expression system applied produces about 33 and 31 mg of purified SAG2-GRA1-ROP1_S_ and SAG2-GRA1-ROP1_L_ proteins from 1 liter of induced culture, respectively. The purification resulted in an electrophoretically homogeneous preparations with a purity above 95 % (results not shown).

### Immunoreactivities of *T. gondii* IgG antibodies in ELISAs with chimeric antigens, mixture of recombinant proteins, and TLA

All of the groups of human serum samples, namely, group I (sera from patients in the acute phase of toxoplasmosis), group II (sera from patients in the postacute phase of toxoplasmosis), group III (sera from patients in the chronic phase of toxoplasmosis), and group IV (sera from seronegative individuals), were examined by means of IgG ELISAs conducted with the SAG2-GRA1-ROP1_S_ and SAG2-GRA1-ROP1_L_ chimeric antigens, a mixture of recombinant antigens (rSAG2, rGRA1, and rROP1), and TLA. Twenty-three sera from group IV (seronegative) were tested in order to calculate cutoff values for all of the IgG ELISAs, which were set as the mean value of the negative serum samples plus 2 standard deviations. Moreover, the remaining 100 sera from this group were used to determine the specificity of the IgG ELISAs. None of these sera reacted above the cutoff value in the IgG ELISA; therefore, the specificity was 100 % (Table 2).

**Table 2:** Comparison of the immunoreactivities of the mixture of antigens (M: rSAG2+rGRA1+rROP1), the SAG2-GRA1-ROP1_S_ and the SAG2-GRA1-ROP1_L_ chimeric antigens, and the TLA using sera from individuals in the acute, postacute, and chronic phase of toxoplasmosis and sera from healthy patients

Serum samples group and antigen	No. of reactive serum samples	Mean absorbance value (range)^a^	Sensitivity [%]	Specificity [%]	PPV^g^ [%]	NPV^h^ [%]
I^b^						
M	41 (100)	2.153 (0.821–3.692)	100	100	100	100
SAG2-GRA1-ROP1S	41 (100)	1.387 (0.293–3.303)	100	100	100	100
SAG2-GRA1-ROP1L	41 (100)	2.143 (0.887–3.626)	100	100	100	100
TLA	41 (100)	1.058 (0.512–1.798)	100	100	100	100
II^c^						
M	17 (100)	0.972 (0.545–1.786)	100	100	100	100
SAG2-GRA1-ROP1S	14 (82.4)	0.529 (0.185–1.431)	82.4	100	100	97.1
SAG2-GRA1-ROP1L	17 (100)	0.963 (0.482–2.184)	100	100	100	100
TLA	17 (100)	0.925 (0.510–1.976)	100	100	100	100
III^d^						
M	113 (99.1)	1.531 (0.466–3.237)	99.1	100	100	99.0
SAG2-GRA1-ROP1S	97 (85.1)	0.593 (0.153–1.713)	85.1	100	100	85.5
SAG2-GRA1-ROP1L	114 (100)	1.403 (0.464–3.148)	100	100	100	100
TLA	112 (98.2)	1.298 (0.460–2.165)	98.2	100	100	98.0
Total^e^						
M	171	1.624 (0.466–3.692)	99.4	100	100	99.0
SAG2-GRA1-ROP1S	152	0.778 (0.153–3.303)	88.4	100	100	83.3
SAG2-GRA1-ROP1L	172	1.535 (0.464–3.626)	100	100	100	100
TLA	170	1.204 (0.460–2.165)	98.9	100	100	98.0
IV^f^						
M	0	0.443 (0.192–0.504)	–	–	–	–
SAG2-GRA1-ROP1S	0	0.166 (0.104–0.279)	–	–	–	–
SAG2-GRA1-ROP1L	0	0.295 (0.109–0.387)	–	–	–	–
TLA	0	0.416 (0.213–0.507)	–	–	–	–

^a^The cutoff values were 0.504 for M, 0.281 for SAG2-GRA1-ROP1_S_, 0.389 for SAG2-GRA1-ROP1_L_, and 0.507 for TLA

^b^Acute phase of toxoplasmosis, *n* = 41

^c^Postacute phase of toxoplasmosis, *n* = 17

^d^Chronic phase of toxoplasmosis, *n* = 114

^e^All of positive serum samples, *n* = 172

^f^Control group negative for anti-*T. gondii* antibodies, *n* = 100

^g^Positive predictive value

^h^Negative predictive value

**Fig. 1:**
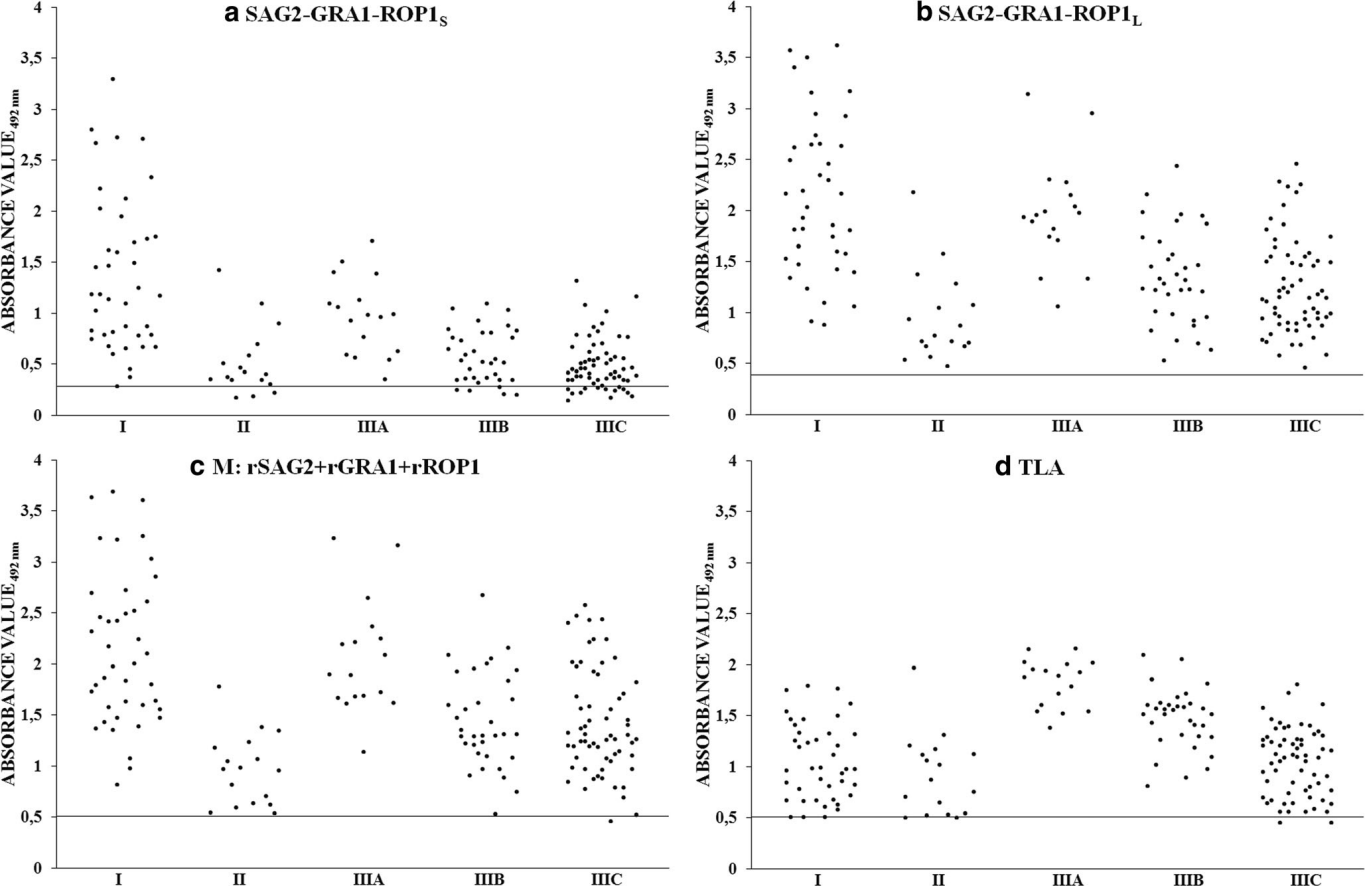
Immunoreactivities of SAG2-GRA1-ROP1_S_ chimeric antigen (**a**), SAG2-GRA1-ROP1_L_ chimeric antigen (**b**), the mixture of recombinant antigens (M: rSAG2+rGRA1+rROP1) (**c**), and TLA (**d**) with sera from patients with suspected acute (I), postacute (II) phase, and chronic (IIIA with IgG value >300 IU/ml, IIIB with IgG value from 101 to 300 IU/ml, and IIIC with IgG value ≤100 IU/ml) phase of toxoplasmosis

The sensitivity of the IgG ELISA with the SAG2-GRA1-ROP1_L_ chimeric antigen (containing longer fragment of ROP1 protein corresponding to amino acid residues 85–396) calculated for all of the positive serum samples was 100 % (Table 2). This value was the highest obtained among the IgG ELISAs and was comparable to IgG ELISAs carried out with the mixture of recombinant antigens (rSAG2, rGRA1, and rROP1), and TLA, whose sensitivity was slightly lower and amounted to 99.4 and 98.8 %, respectively. Much lower sensitivity at 88.4 % was obtained for the IgG ELISA with the SAG2-GRA1-ROP1_S_ chimeric antigen (which contained a shorter fragment of ROP1 protein corresponding to amino acid residues 85–250). Examination of the results obtained for serum samples from patients in the acute phase of toxoplasmosis (group I) showed that the IgG antibodies reacted at 100 % with a mixture of recombinant protein (rSAG2, rGRA1, and rROP1), the SAG2-GRA1-ROP1_S_ and the SAG2-GRA1-ROP1_L_ chimeric antigens, and the TLA. In group II consisting of sera from patients in the postacute phase of toxoplasmosis, specific IgG antibodies reacted from all of the serum samples in the case of using the SAG2-GRA1-ROP1_L_ chimeric antigen, the mixture of recombinant proteins and the TLA (100 %), whereas the reactivity of the IgG antibodies with the SAG2-GRA1-ROP1_S_ chimeric antigen was lower, at 82.4 %. Moreover, sera from the patients in the chronic phase of toxoplasmosis, group III, reacted with the SAG2-GRA1-ROP1_L_ chimeric antigen with the highest reactivity of 100 %, whereas slightly lower reactivity was observed for the mixture of recombinant antigens and the TLA, at 99.1 and 98.2 %, respectively. As in the case of group II, the lowest reactivity at 85.1 % was noticed for the SAG2-GRA1-ROP1_S_ chimeric antigen. Additionally, the results obtained for serum samples of group III were divided into subgroups for the IgG antibody titers. Thus, all of the serum samples from subgroup IIIA (IgG antibodies value >300 IU/ml) reacted at 100 % with a mixture of antigens, the tested chimeric antigens, and the TLA (Fig. 1). The same reactivity was observed for a mixture of recombinant proteins, the SAG2-GRA1-ROP1_L_ chimeric antigen, and TLA with sera from subgroup IIIB (IgG antibodies value between 101 and 300 IU/ml), whereas the reactivity was much lower for the SAG2-GRA1-ROP1_S_ chimeric antigen, at 85.3 %. In the case of subgroup IIIC (IgG antibodies value ≤100 IU/ml), the reactivity at 100 % was observed for the SAG2-GRA1-ROP1_L_, whereas slightly lower reactivity was observed for a mixture of recombinant antigens and the TLA, at 98.4 and 96.8 %, respectively. The lowest reactivity at 81.0 % was noticed for the SAG2-GRA1-ROP1_S_ chimeric antigen.

## Discussion

Until now, there were only a few studies demonstrating the usefulness of the recombinant chimeric antigens in the detection of specific anti-*T. gondii* antibodies. In 2006, Baghetto et al. showed the usefulness of two chimeric antigens in serological diagnosis of toxoplasmosis. One of those contained three immunodominant regions of MIC2, MIC3, and SAG1; the second one contained three fragments of GRA3, GRA7, and M2AP. In 2011, Lau et al. constructed a chimeric antigen which was composed of the SAG1 and SAG2 fragments. The usefulness of this chimeric antigen in Western blot assays using serum samples from patients with early acute, acute, and chronic toxoplasmosis was shown. Dai et al. (2012, 2013) developed a recombinant multiepitope fusion peptide (rMEP) composed of three antigenic determinants cloned from SAG1, SAG2, and SAG3 antigens which was effective in discriminating between active and chronic infections and useful for the detection of IgG and IgM antibodies. In our previous studies, we also showed the usefulness of two recombinant chimeric antigens MIC1-MAG1 (Holec-Gąsior et al. 2012a) and MIC1-MAG1-SAG1 (Holec-Gąsior et al. 2012b) in diagnosis of toxoplasmosis. We demonstrated that the single recombinant proteins MIC1ex2 and MAG1 or mixtures of proteins (MIC1ex2 and MAG1) have lower reactivity at the level of 75.5, 60, and 69.1 %, respectively, than the polyvalent native antigen TLA at 91.8 %. Only the chimeric antigen MIC1-MAG1 had a comparable reactivity to the TLA, which amounted to 90.9 %. Furthermore, in the case of serum samples from patients with the acute and postacute phase of toxoplasmosis the MIC1-MAG1 chimeric antigen was characterized by 100 % reactivity, while the TLA showed lower reactivity at 88.5 %. In the next study (Holec-Gąsior et al. 2012b), we showed that the MIC1-MAG1-SAG1 chimeric protein containing three *T. gondii* antigen fragments had higher reactivity than the chimeric antigen MIC1-MAG1, which contained only two protein fragments. The addition to the chimeric protein of the fragment of SAG1, which is one of the most immunogenic parasite proteins, resulted in an increase in the reactivity with specific IgG anti-*T. gondii* antibodies. The reactivity of the IgG ELISA with the MIC1-MAG1-SAG1 chimeric antigen calculated for all of the positive serum samples was 98.1 %, while the sensitivity of the IgG ELISA with the MIC1-MAG1 chimeric antigen or mixture of three recombinant proteins (rMIC1ex2, rMAG1, and rSAG1) was significantly lower and amounted 81.5 and 90.7 %, respectively. The result of our studies showed that in the case of antigen construction for diagnostic utility, a rational selection of protein fragments is of great importance. The above described studies show the potential of recombinant chimeric antigens. Major advantages of the recombinant chimeric antigens over commonly tested individual recombinant antigens and/or mixture of those include (i) higher sensitivity of immunoassay tests and (ii) simplicity to define a quality of the chimeric antigen and to standardize the method.

In the present study, further work on the utility of the recombinant chimeric antigens in IgG ELISA tests for human toxoplasmosis diagnosis is shown. Three fragments of *T. gondii* SAG2, GRA1, and ROP1 proteins were used to construct two chimeric antigens which contain the same fragment of SAG2 and GRA1 antigens, but a different sizes fragment of the ROP1 protein. The SAG2-GRA1-ROP1_S_ and SAG2-GRA1-ROP1_L_ chimeric antigens were evaluated with the IgG ELISA. The study demonstrates that, in all of the positive sera, the sensitivity of IgG ELISA for SAG2-GRA1-ROP1_L_ chimeric antigen (contains 85–396 amino acid residues of ROP1) was 100 %. The reactivity of the IgG ELISA for the mixture of proteins (rSAG2, rGRA1, and rROP1), and TLA was almost as high as for the SAG2-GRA1-ROP1_L_, at 99.4 and 98.8 %, respectively. Significantly lower reactivity at 88.4 % was reported for IgG ELISA with the use of the SAG2-GRA1-ROP1_S_ chimeric antigen (contains 85–250 amino acid residues of ROP1). Moreover, the specific IgG anti-*T. gondii* antibodies in individual sera from patients with acute phase of toxoplasmosis reacted at the same level with all antigen preparations. In group II (sera from patients with postacute phase of toxoplasmosis), lower reactivity at 82.4 % was noticed only for SAG2-GRA1-ROP1_S_ chimeric protein. The examination of the sera from group III, collected from patients with the chronic phase of toxoplasmosis demonstrated that the sensitivity of IgG ELISA is associated with IgG titers. Accurate analysis of the serum samples from this group showed that only the reactivity of the IgG ELISA with the SAG2-GRA1-ROP1_L_ chimeric antigen is still high, at 100 %, for sera from all subgroups (IIIA, IIIB, and IIIC). Furthermore, a slightly lower reactivity, at 98.4 and 96.8 %, was noticed properly of the IgG ELISA with the mixture of antigens and the TLA, for sera from patients with a low IgG value of ≤100 IU/ml in IIIC subgroup (IgG value of nonreactive serum samples at ≤15 IU/ml). The lowest reactivity was again observed in the case of the IgG ELISA with the SAG2-GRA1-ROP1_S_ chimeric antigen, in subgroup IIIB (IgG value between 101 and 300 IU/ml) at 85.3 % and for subgroup IIIC (IgG value of ≤100 IU/ml) at 81.0 %. Moreover, the mean absorbance values calculated for all three groups of serum samples showed that the SAG2-GRA1-ROP1_L_ chimeric antigen, and a mixture of three recombinant proteins (rSAG2, rGRA1, and rROP1) resulted in comparably higher reactivity than TLA, and the SAG2-GRA1-ROP1_S_ chimeric antigen.

The constructed chimeric antigens contain fragments of three *T. gondii* proteins, SAG2, and GRA1 characterized by the ability to detect IgG antibodies in sera from both acute and chronic toxoplasmosis (Hiszczyńska-Sawicka et al. 2003, 2005; Lau and Fong 2008; Lecordier et al. 2000; Li et al. 2000; Parmley et al. 1992; Pietkiewicz et al. 2007), and ROP1, which has been suggested to be a molecular marker to differentiate between acute and chronic infection (Holec-Gąsior et al. 2009, 2010). The higher reactivity of the SAG2-GRA1-ROP1_L_ chimeric antigen (contains 85–396 amino acid residues of ROP1) than the SAG2-GRA1-ROP1_S_ chimeric antigen (contains 85–250 amino acid residues of ROP1) can be explained by the fact that the only construct containing a bigger fragment of the ROP1 protein was recognized by specific IgG anti-*T. gondii* antibodies targeted for the ROP1 antigen. Furthermore, the absorbance values observed for individual serum samples is a confirmation of this fact, the average values of the absorbance was twice as high for the SAG2-GRA1-ROP1_L_ chimeric antigen than for the SAG2-GRA1-ROP1_S_ chimeric antigen. It is possible that the shorter fragment of the ROP1 antigen is not as well recognized by specific IgG antibodies, and absorbance values derived from the antibodies specifically bound to the fragments of SAG2 and GRA1 antigens. This hypothesis is supported by the results where the mean absorbance values obtained for the mixture of antigens (rSAG2, rGRA1, and rROP1) and the SAG2-GRA1-ROP1_L_ chimeric antigen were almost the same.

The study of the ROP1 antigen showed that the amino acid sequence of these proteins contains an interesting charge asymmetry suggesting a role in the heterotypic binding of different proteins (Bradley and Boothroyd 1999; Ossorio et al. 1992; Soldati et al. 1995). The mature ROP1 protein has a series of tandem octapeptide repeats rich in proline and glutamic acid (gives the acidic character of N-terminal region and center of protein), the antigen also contains a region rich in arginine (alkaline region in the C-terminal), which may stabilize the structure of the protein. The SAG2-GRA1-ROP1_L_ chimeric antigen with a fragment of ROP1 corresponding to 85–396 amino acid residues, contains a part of the alkaline C-terminal region rich in arginine, which is likely to be crucial for antigen recognition by the specific antibodies. The high reactivity of the SAG2-GRA1-ROP1_L_ chimeric antigen can be also explained by another hypothesis. The C-terminal region of ROP1 may contain at least one immunodominant B-cell epitope recognized by the majority of individuals, whereas responses to N-terminal region of ROP1 are much more variable. In 2000, Lecordier et al. showed that only the N-terminal hydrophilic part of the GRA6 protein was recognized by a pool of positive human sera in an immunoblot, while the same serum samples did not yield positive results with the C-terminal part of GRA6. In 2008, Holec et al. observed different reactivity values of serum samples from patients with acute and chronic infection in the IgG ELISA with the MIC1ex2 (N-terminal part of MIC1), the MIC1ex34 (C-terminal part of MIC1), and the whole MIC1. The result also showed that only the N-terminal part of MIC1 (rMIC1ex2) reacts strongly with both groups of sera from acute (96.1 %) and chronic (75 %) phase of toxoplasmosis. These results demonstrated that for some antigens the reactivity of specific IgG antibodies was correlated with a particular part of the antigen, and probably the same situation occurred for the ROP1 protein. Moreover, the results obtained in this study confirm the results from our previous studies, which indicated that a properly constructed chimeric antigen containing different immunodominant regions is better than a mixture of antigens and can be used instead of TLA in serodiagnosis of human toxoplasmosis.

To summarize, this report presents results which demonstrate, for the first time, that during the construction of chimeric antigens in addition to a rational choice of proteins which exhibit strong immunogenic properties, particular attention should be also paid to the size of protein fragments and its amino acid sequences. Furthermore, these studies show the diagnostic usefulness of the new SAG2-GRA1-ROP1_L_ and SAG2-GRA1-ROP1_S_ chimeric antigens. One of these antigens SAG2-GRA1-ROP1_L_ in the IgG ELISA was characterized by a significantly higher reactivity/sensitivity than that obtained with the TLA or with a mixture of three antigens. Before the SAG2-GRA1-ROP1_L_ chimeric antigen can be used instead of the TLA in the clinical diagnosis of toxoplasmosis more assays are required; however, the presented results are very promising.
